# Investigation of the presence and antinociceptive function of muscarinic acetylcholine receptors in the African naked mole-rat (*Heterocephalus glaber*)

**DOI:** 10.1007/s00359-015-1048-x

**Published:** 2015-10-31

**Authors:** Kristine B. Jørgensen, Karen Krogh-Jensen, Darryl S. Pickering, Titus I. Kanui, Klas S. P. Abelson

**Affiliations:** Department of Experimental Medicine, Faculty of Health and Medical Sciences, University of Copenhagen, Blegdamvej 3B, 2200 Copenhagen, Denmark; Department of Drug Design and Pharmacology, Faculty of Health and Medical Sciences, University of Copenhagen, Universitetsparken 2, 2100 Copenhagen, Denmark; School of Agricultural and Veterinary Sciences, South Eastern Kenya University, P.O. BOX 170-90200, Kitui, Kenya

**Keywords:** Naked mole-rat, Cholinergic receptors, Muscarinic, antinociception, formalin test

## Abstract

The present study investigated the cholinergic system in the African naked mole-rat (*Heterocephalus glaber*) with focus on the muscarinic acetylcholine receptor subtypes M_1_ and M_4_. The protein sequences for the subtypes *m*_*1–5*_ of the naked mole-rat were compared to that of the house mouse (*Mus musculus*) using basic local alignment search tool (BLAST). The presence and function of M_1_ and M_4_ was investigated in vivo, using the formalin test with the muscarinic receptor agonists xanomeline and VU0152100. Spinal cord tissue from the naked mole-rat was used for receptor saturation binding studies with [^3^H]-N-methylscopolamine. The BLAST test revealed 95 % protein sequence homology showing the naked mole-rat to have the genetic potential to express all five muscarinic acetylcholine receptor subtypes. A significant reduction in pain behavior was demonstrated after administration of 8.4 mg/kg in the formalin test. Administration of 50 mg/kg VU0152100 resulted in a non-significant tendency towards antinociception. The antinociceptive effects were reversed by the muscarinic acetylcholine receptor antagonist atropine. Binding studies indicated presence of muscarinic acetylcholine receptors with a radioligand affinity comparable to that reported in mice. In conclusion, muscarinic acetylcholine receptor subtypes are present in the naked mole-rat and contribute to antinociception in the naked mole-rat.

## Introduction

Chronic pain is a major challenge in pain research and medicine, since it is often multifactorial and very difficult to treat (Pergolizzi et al. 2012). Novel treatment strategies against chronic pain are desirable, and in the search for such treatments, the use of experimental animal models is necessary.

The involvement of the muscarinic cholinergic receptor system in antinociception is well established. Five muscarinic receptor subtypes have been identified so far, denominated M_1_–M_5_ (based on pharmacological characterization) or *m*_*1*_–*m*_*5*_ (based on genes coding for the receptors) (Caulfield and Birdsall 1998). Antinociceptive effects of cholinomimetic drugs, such as oxotremorine (George et al. 1962; Harris et al. 1969; Ireson 1970; Bartolini et al. 1987), pilocarpine (Hendershot and Forsaith 1959), physostigmine (Harris et al. 1969; Ireson 1970), tremorine, arecoline and diisopropylphosphorofluoridate (DEP) have been investigated in different laboratory animal species (Bartolini et al. 2011). Muscarinic receptor subtypes interact with other receptor systems in the spinal cord including the GABAergic, opioid, serotonergic and adrenergic systems (Baba et al. 1998; Li and Zhuo 2001; Chen and Pan 2003; Honda et al. 2003; Abelson and Höglund 2004; Chen and Pan 2004; Kommalage and Höglund 2005b; Kommalage and Höglund 2005a). In addition, cholinergic antagonists have been shown to block antinociception in rats (Abelson and Höglund 2002).

The muscarinic receptor subtype M_1_ is involved in neuronal activity and analgesia (Bartolini et al. 2011; Martino et al. 2011), and is highly expressed in the brain, but not the spinal cord of rats (Höglund and Baghdoyan 1997; Bartolini et al. 2011), while presence in human spinal cord has been suggested (Villiger and Faull 1985).

The M_4_ receptor subtype is highly concentrated in the superficial layers of the spinal cord dorsal horn, in the brain of both rats and humans (Schechtmann et al. 2008), and in high levels in the striatum (Levey et al. 1991; Levey 1993). The M_4_ receptors are considered important in muscarinic-mediated analgesia together with the M_2_ subtype (Ellis et al. 1999; Zhang et al. 2002; Chen and Pan 2004; Martino et al. 2011). The suggested antinociception-mediating effects and for the muscarinic receptor subtypes M_1_ and M_4_ makes them interesting potential drug targets for pain relief, but there is still a considerable lack of knowledge about the specific mechanism by which the muscarinic receptors exert their antinociceptive effect. By undertaking comparative studies of the muscarinic cholinergic receptor system in different species, we strive to gather important information about the antinociceptive functions of this system that would contribute to an increased understanding of cholinergic antinociceptive mechanisms.

The African naked mole-rat (*Heterocephalus glaber*) possesses several unusual features compared to other rodents, including a pain system with several deviations from the pain system of other mammals (Kanui et al. 1993; Towett and Kanui 1993; Towett et al. 2006, 2009; Park et al. 2008; Kim et al. 2011). It has recently been established as a model for studying cholinergic involvement in pain behavior (Dulu et al. 2014). Thus, it is possible to investigate whether the difference in pain behavior in the naked mole-rat is related to differences in the presence and function of muscarinic receptors, compared to other species.

The aim of the present study was to confirm the presence and function of muscarinic receptors, in particular the M_1_ and M_4_ subtypes, in the naked mole-rat, using the formalin test and saturation binding studies on spinal cord tissue. It was hypothesized that the muscarinic receptor subtypes M_1_ and M_4_ contribute to the regulation of pain transmission in the naked mole-rat, similar to commons rodent species used in biomedical pain research.

## Materials and methods

### Animals

African naked mole-rats were captured under a permit issued by the Kenya Wildlife Services (KWS). The experimental procedures were performed after ethical approval of KWS and of the Institutional Animal Care and Use Committee (the Faculty of Veterinary Medicine Research Ethics Committee of the University of Nairobi). The experimental procedures were conducted in accordance with the guidelines set forth by the American Physiological Society (American Physiological Society 2002).

The mole-rats were captured from Kathekani, Makueni County in Southeastern Kenya, during the months of July–August. A total of 100 animals of both sexes were captured, varying in mass (12.9–54.7 g) and age. Nineteen animals died of unknown causes from the time of capture until the experiment was initiated. The animals were allowed a 1-month acclimatization period in the laboratory.

For the receptor binding studies, ten naked mole-rats were euthanized by cervical dislocation and the spinal cords were removed. None of these animals had undergone any pharmacological treatments.

### Housing

The animals were housed in one housing unit consisting of four plastic barrels; two measured 46 cm in diameter and 30 cm tall and two measured 39 cm in diameter and 36 cm tall. The barrels were connected with plastic tubing to mimic the burrows. The ambient temperature was kept at 25 ± 2 °C and the relative humidity at 45–50 %. The bedding consisted of coarse sawdust and was changed as needed. The animals were fed on fresh carrots and sweet potatoes ad libitum and no water was supplied.

### Basic local alignment search tool (BLAST) analysis

The accession numbers for the amino acid sequences encoded by *m*_1_–*m*_5_ in the house mouse (*Mus musculus*) and in the naked mole-rat were found via the National Center for Biotechnology Information. A BLAST comparison was made on the basis of each of the accession numbers and the maximal identity, query cover, maximal score and total score to a similar sequence in the naked mole-rat genome was noted.

### Formalin test

The in vivo formalin tests on the African naked mole-rats were performed at South Eastern Kenya University, Kitui, Kenya. Thirty-seven animals were injected subcutaneously (s.c.) with 20 μL of 3.7 % (w/v) formalin or 0.9 % (w/v) NaCl (saline vehicle) dorsally in the right hind paw using a 27G needle and a 1 ml syringe, based on a previous study (Towett et al. 2009), and placed in a glass observation chamber measuring 15 × 14.5 × 14.5 cm. Animals were observed immediately after injection and the time spent licking, biting or flinching the injected paw were recorded in blocks of 5 min during a total of 60 min. The observer was blinded to the formalin or vehicle treatments. During experiments with muscarinic receptor ligands, the animals were injected with 20 μL formalin s.c. dorsally in the right hind paw 30 min after the intraperitoneal (i.p.) injection of ligand. Immediately after formalin injection, the animal was placed in the observation chamber for an hour and observed as described above. After each test the animal’s motor skills and proprioception were checked. This was done by placing the animal on its back to see if it could turn over and also by placing the dorsal surface of each hind paw on the table to see if it could sense this and turn it over. The majority of the experiments were performed between 8 a.m. and 5 p.m. at a room temperature of 25.7–27.8 °C.

### Receptor saturation binding

#### Tissue

Whole spinal cords from ten euthanized naked mole-rats were immediately removed and stored in a plastic container, submerged in 0.9 % ice-cold NaCl. The tissue was stored at −20 °C. Upon arrival at the University of Copenhagen, tissues were stored at −80 °C. On the day of the experiment, the containers were slowly thawed by placing the container in cold water. The spinal cords were dissected from the vertebrae in a petri dish kept on ice. The dissection was done by exposing the vertebrae and cutting through the vertebrae with scissors. The spinal cord was lifted out from the vertebrae with forceps, and dorsal roots were cut and discarded to avoid possible contamination from dorsal root ganglia. Spinal cords were pooled in tubes and weighed so each contained a minimum of 200 mg. Since the tissue available was limited, it was not possible to isolate the lumbar region alone, although this region could be assumed to correspond to the behavioral tests conducted. The tubes were kept frozen at −80 °C until used for binding experiments.

Spinal cord tissue (200–300 mg) was thawed and homogenized at 24,000 rpm for 1 min in 50 volumes of ice-cold 30 mM Na^+^/HEPES wash buffer (pH 7.5 at 4 °C) using an Ultra-Turrax (IKA, Staufen, Germany) homogenizer and then centrifuged at 48,000×*g* for 20 min at 4 °C. The resulting pellet was resuspended in 50 volumes of ice-cold wash buffer and re-centrifuged. The washed pellet was finally resuspended in 50 volumes of 30 mM Na +/HEPES assay buffer (pH 7.5 at room temperature). Protein concentration of the membrane suspension was determined using the micro-BCA protein assay (Thermo Fisher Scientific, Rockford, IL, USA).

#### Saturation binding

Saturation binding of [^3^H]-N-methylscopolamine ([^3^H-NMS)] (85.5 Ci/mmol; PerkinElmer, Waltham, MA, USA) to spinal cord membranes was carried out in triplicate in glass tubes for 1 h at room temperature in a volume of 0.25 ml using 0.01–4.0 nM radiolabel. Non-specific binding at each radiolabel concentration was determined using 1 µM atropine. The binding reaction was terminated by adding 3 ml of ice-cold wash buffer and rapid vacuum filtration through GF/B glass fiber filters (VWR International, Denmark) pre-soaked for 1 h in 0.3 % (w/v) polyethylenimine/50 mM Tris base. Filters were immediately rinsed once with 3 ml ice-cold wash buffer. Tissue-bound radioactivity was extracted from the filters by overnight immersion in 3 ml EcoScint A scintillation fluid (National Diagnostics, Atlanta, GA, USA) and radioactivity was measured as DPM by liquid scintillation counting.

### Drugs and chemicals

Xanomeline was chosen because of its properties as muscarinic receptor agonist selective for M_1_ and M_4_ (Martino et al. 2011). VU152100 was chosen because of its properties as positive allosteric modulator of muscarinic receptor M_4_ (Brady et al. 2008).

Xanomeline l-tartrate hydrate, VU0152100, and atropine sulfate were obtained from Sigma–Aldrich, Denmark. Xanomeline and atropine were dissolved in 0.9 % (w/v) saline and stored at 2–4 °C. VU0152100 was dissolved in 20 % (v/v) DMSO and stored at 2–4 °C.

### Data analysis

#### Formalin test

The data were analyzed using two-way ANOVA with Bonferroni’s post hoc test in GraphPad Prism version 5.0. Data are presented as mean time (±SEM) in graphs. *P* < 0.05 was considered statistically significantly different.

#### Saturation binding

Receptor saturation binding data were analyzed in GraphPad Prism version 5.0 using non-linear regression, saturation binding, one site, total + non-specific binding, robust fit to determine the *K*_d_ and *B*_max_ values and using non-linear regression, one site specific binding to determine the Hill coefficient, *n*_H_.

## Results

### Blast

Using GenBank data from the National Center for Biotechnology Information (NCBI), the accession numbers and amino acid sequences encoded by *m*_1_–*m*_5_ in the house mouse (*Mus musculus*) and in the naked mole-rat were obtained. Table 1 shows the results from comparing the amino acid sequences of mAChRs in the house mouse with the sequences of mAChRs in the naked mole-rat via BLAST.

**Table 1:** An overview of the results from comparing gene sequences of mAChRs in the house mouse with protein sequences of mAChRs in the African naked mole-rat

	Maximal score	Total score	Query cover (%)	Maximal identity (%)
*m*_*1*_ *Mus musculus* NP_001106167/*m*_*1*_ *Heterocephalus glaber* EHB02109	652	652	81	95
*m*_*2*_ *Mus musculus* NP_987076/*m*_*2*_ *Heterocephalus glaber* EHA98116	891	891	97	97
*m*_*3*_ *Mus musculus* NP_150372/*m*_*3*_ *Heterocephalus glaber* EHB16914	1101	1101	100	92
*m*_*4*_ *Mus musculus* NP_031725/*m*_*4*_ *Heterocephalus glaber* EHB00279	903	903	100	95
*m*_*5*_ *Mus musculus* NP_991352/*m*_*5*_ *Heterocephalus glaber* EHA97883	959	959	100	89

The first column shows accession numbers for the protein sequences used to compare

### Formalin test

#### Control study

**Fig. 1:**
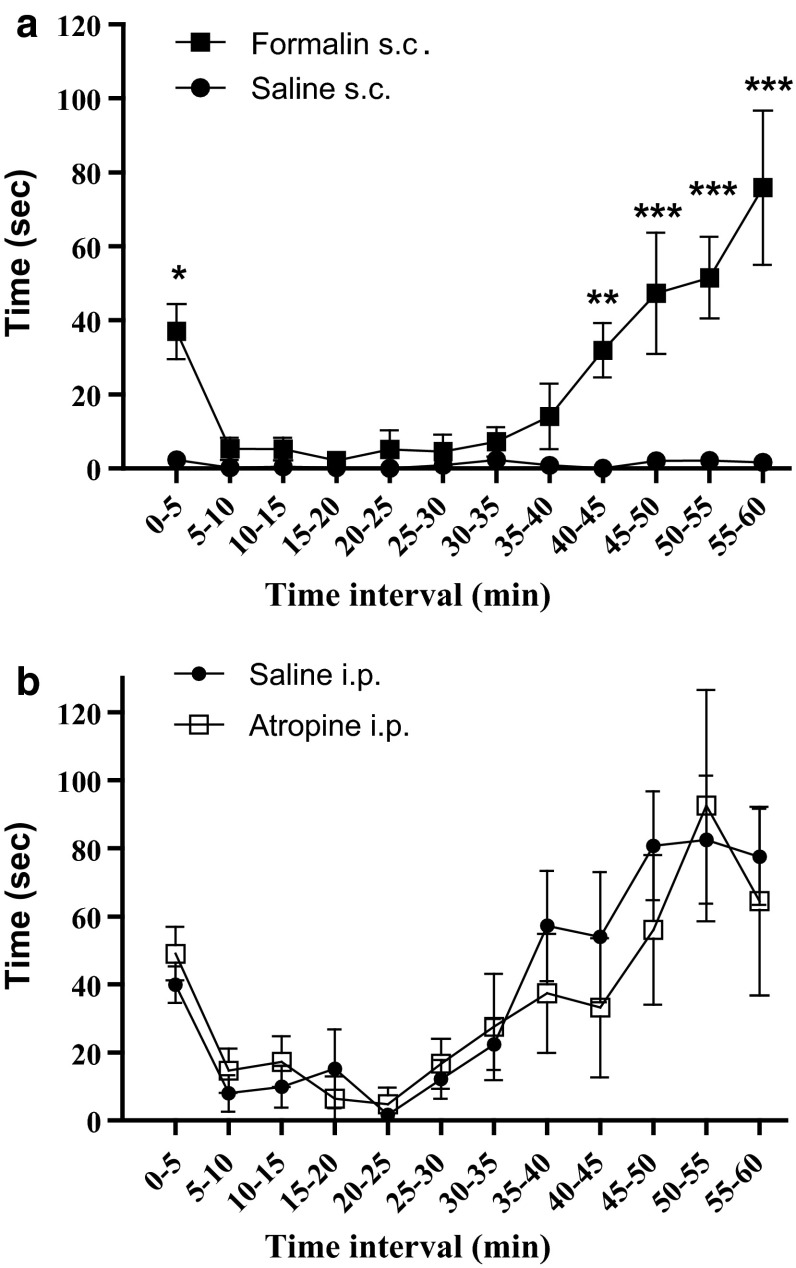
**a** The effect of subcutaneous injection of 20 μl 10 % formalin (*filled square*) or saline (*filled circle*) dorsally in the right hind paw on pain behavior. A significant difference was found in the time intervals 0–5 min (*P* < 0.01), 40–45 min (*P* < 0.05), and 45–60 min (*P* < 0.001). Data are shown as mean (±SEM). Number of animals (*n*) = 8 for saline, *n* = 8 for formalin. Data were analyzed by two-way ANOVA with Bonferroni’s post hoc test demonstrated using GraphPad Prism 5.0. *P < 0.01, **P < 0.05, *P < 0.001. **b** A comparison of the injection of i.p. saline (*filled circle*) and i.p. atropine (*unfilled rectangle*; 2.5 mg/kg). No significant difference (*P* > 0.05) was found in the late or early phase. Data are shown as mean time (±SEM). Number of animals (*n*) = 8 for saline and *n* = 5 for atropine. Data were analyzed by two-way ANOVA and Bonferroni’s post hoc test using GraphPad Prism 5.0

There was a significant difference in pain behavior between injection of 20 μL 10 % formalin and saline s.c. in the time intervals 0–5 min (*P* < 0.01), 40–45 min (*P* < 0.05), and 45–60 min *(P* < 0.001) (Fig. 1). No deterioration in proprioception or motor skills was observed. On the basis of the biphasic appearance of the graph (Fig. 1) and the significant differences between the formalin and saline injections, the early and late phase were determined to be 0–10 and 35–60 min, respectively. Although there were no significant differences during the 5–10 and 35–40 min intervals, they were included due to observed pain behavior during the 5–10 min interval and the slight incline beginning from 35 min. All results for the experiments of control groups, xanomeline and VU0152100 were found to follow a Gaussian distribution, as determined with D’Agostino and Pearson omnibus normality test.

#### Xanomeline

The effect of intraperitoneal (i.p.) injection of xanomeline in doses of 0.84, 2.8, 8.4 and 28.1 mg/kg was investigated and was compared to a control group receiving i.p. injection of saline. Doses were based on the study by Martino et al. (2011). Xanomeline 28.1 mg/kg was found to affect motor functions in the animals. Two out of four animals had no proprioception and could not turn over when placed on their backs. The third animal had reduced proprioception and could turn over from its back while the fourth had normal proprioception and motor skills. Since this makes it impossible to distinguish an antinociceptive effect from immobilization, the highest dose of xanomeline was omitted from the study.

**Fig. 2:**
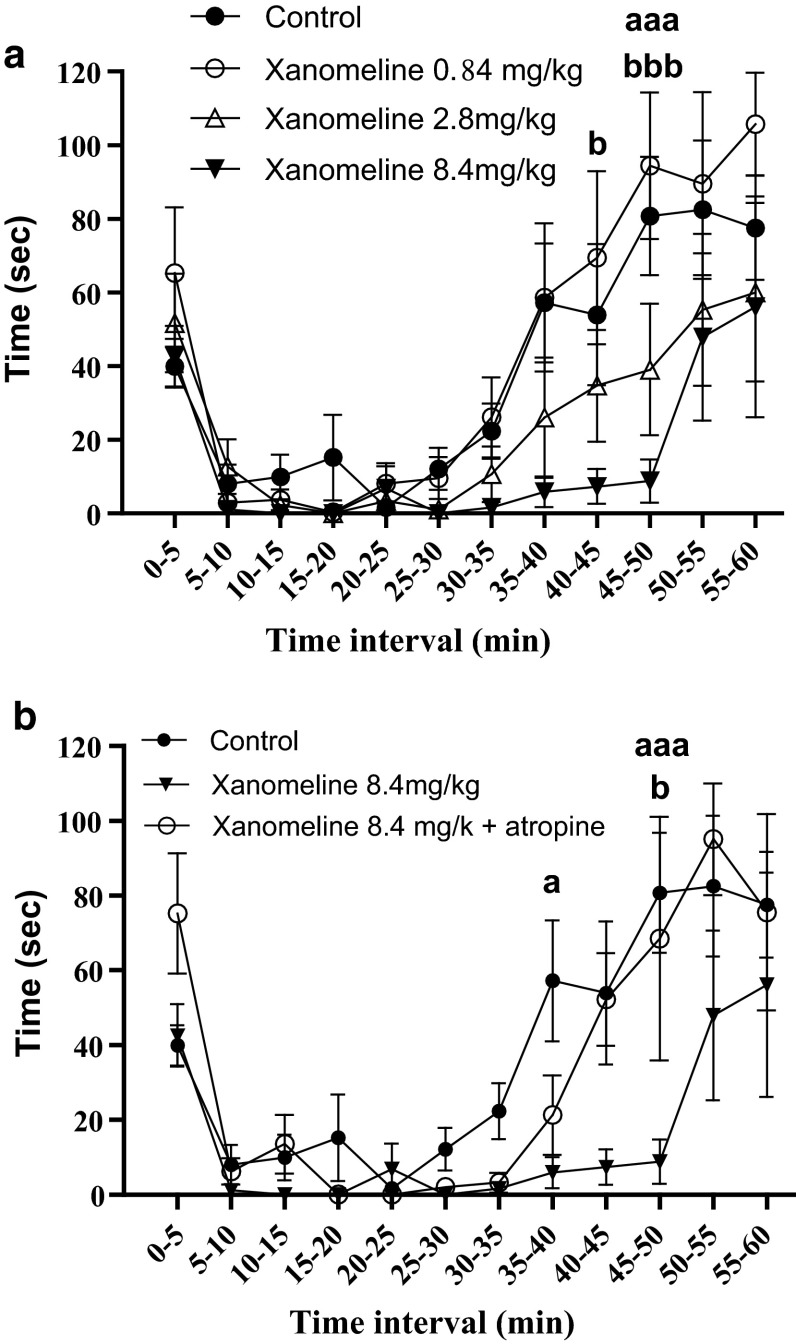
**a** The effect of i.p. injection of xanomeline in concentrations of 0.84 (*unfilled circle*), 2.8 (*unfilled up pointed triangle*) and 8.4 (*filled down pointed triangle*) mg/kg compared to a control group (*filled circle*) of i.p. saline injection. A significant difference was found in the late phase during the time interval of 45–50 min (*P* < 0.001) when comparing the injection of xanomeline in the concentration of 8, 4 mg/kg to the control group, indicated with *aaa*. A significant difference was also found when comparing the injection of xanomeline at the concentration of 0.84 mg/kg to xanomeline in the concentration of 8, 4 mg/kg during the time intervals of 40–45 min (*P* < 0.05) and 45–50 min (*P* < 0.001), indicated with *b* and *bbb*, respectively. Data are shown as mean time (±SEM). Number of animals (*n*) = 8 for control, *n* = 6 for xanomeline 0.84 mg/kg, *n* = 6 for xanomeline 2.8 mg/kg, *n* = 6 for xanomeline 8.4 mg/kg. Data were plotted using GraphPad Prism 5.0. **b** The effect of i.p. injection of xanomeline 8.4 (*unfilled circle*) mg/kg co-administered with atropine (2.5 mg/kg), compared to the i.p. injection of 8.4 (*filled down pointed triangle*) mg/kg xanomeline alone and the saline (*filled circle*) control group. Significant differences were found in the late phase during the time intervals 35–40 min (*P* < 0.05) and 45–50 min (*P* < 0.0001) when comparing the injection of xanomeline 8.4 mg/kg alone with the control group, indicated with *a* and *aaa*, respectively. A significant difference was also found in the time interval 45–50 min when comparing the injection of xanomeline 8.4 mg/kg alone with xanomeline 8.4 mg/kg co-administered with atropine (*P* < 0.05), indicated with *b*. No significant difference was found between the control group and xanomeline 8.4 mg/kg co-administered with atropine. Data are shown as mean time (±SEM). Number if animals (*n*) = 8 for saline, *n* = 6 for xanomeline 8.4 mg/kg and *n* = 5 for xanomeline 8.4 mg/kg + atropine. Data were analyzed by two-way ANOVA and Bonferroni’s post hoc test using GraphPad Prism 5.0

Results from the control saline group were compared to all xanomeline doses and the different xanomeline doses were compared to each other, in the 5 min time intervals during the early (0–10 min) and late (35–60 min) phases. Figure 2a illustrates the relationship between all the xanomeline doses and the control group. The xanomeline doses of 0.84 and 2.8 mg/kg had no significant effect compared to the control group. I.p. injection of xanomeline 8.4 mg/kg produced a significant decrease in pain behavior compared to the control group in the late phase, at 45–50 min (*P* < 0.001). When comparing this concentration to the other xanomeline doses, a significant difference to 0.84 mg/kg was found during the late phase in the time intervals 40–45 min (*P* < 0.05) and 45–50 min (*P* < 0.001). No deterioration in proprioception or motor skills was observed after administration of 0.84–8.4 mg/kg. i.p. injection of atropine 2.5 mg/kg alone was compared to that of the control i.p. saline injection (Fig. 1b). The atropine dose was based on the study by Dulu et al. (2014). No significant difference was found (*P* > 0.05). No deterioration in proprioception or motor skills was observed. Pain behavior after co-administration of 8.4 mg/kg xanomeline with 2.5 mg/kg atropine is shown in Fig. 2b. A significant difference was found in the late phase at 45–59 min when comparing the effect of atropine and xanomeline 8.4 mg/kg with xanomeline 8.4 mg/kg alone. No deterioration in proprioception or motor skills was observed.

#### Vu0152100

**Fig. 3:**
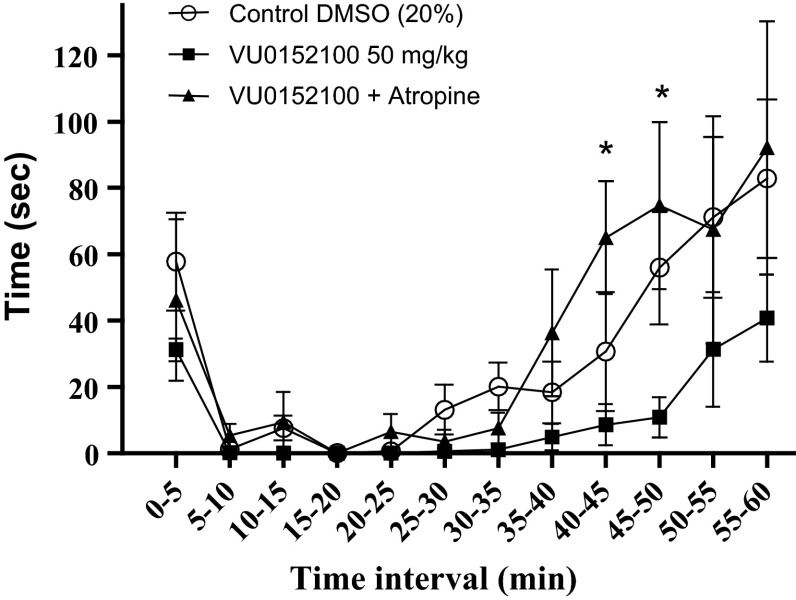
The effect of i.p. injections of the control group of 20 % DMSO (*unfilled circle*), 50 mg/kg V0152100 (*filled square*) and co-administration of 50 mg/kg VU0152100 with 2.5 mg/kg atropine (*filled up pointed triangle*). No significant reduction in pain behavior (*P* > 0.05) was found when comparing IP injection of VU0152100 with the i.p. injection of DMSO 20 % control group, or with VU0152100 and co-administration of VU0152100 with atropine. A significant difference was found between i.p. injections of VU0152100 and co-administration of VU0152100 with atropine, in the late phase during the time intervals of 40–50 min (*P* < 0.05). Data are shown as mean time (±SEM). Number of animals (*n*) = 6 for DMSO 20 %, *n* = 6 for VU0152100 and *n* = 4 for co-administration of VU0152100 with atropine. Data were analyzed by two-way ANOVA and Bonferroni’s post hoc test using GraphPad Prism 5.0. *P < 0.05

Since VU0152100 was dissolved in 20 % (v/v) DMSO, a control group of DMSO was compared to the i.p. saline control group. No significant difference was found (*P* > 0.05), and thus the DMSO group was used as a control group for the VU0152100 experiments. The effect of i.p. injection of VU0152100 in a concentration of 50 mg/kg was investigated and the mean time (±SEM) spent showing pain behavior is shown in Fig. 3. The dose of VU152100 was based on the study by Brady et al. (2008). No significant reduction in pain behavior (*P* > 0.05) was found when compared with the DMSO control group, although a tendency was observed. No deterioration in proprioception or motor skills was observed.

The mean time (±SEM) spent showing pain behavior after i.p. co-administration of 50 mg/kg VU0152100 with 2.5 mg/kg atropine is shown in Fig. 3. A significant difference was found in the late phase (*P* < 0.05) when compared to the i.p. injection of just 50 mg/kg VU0152100. No deterioration in proprioception or motor skills was observed.

### Receptor saturation binding assay

**Fig. 4:**
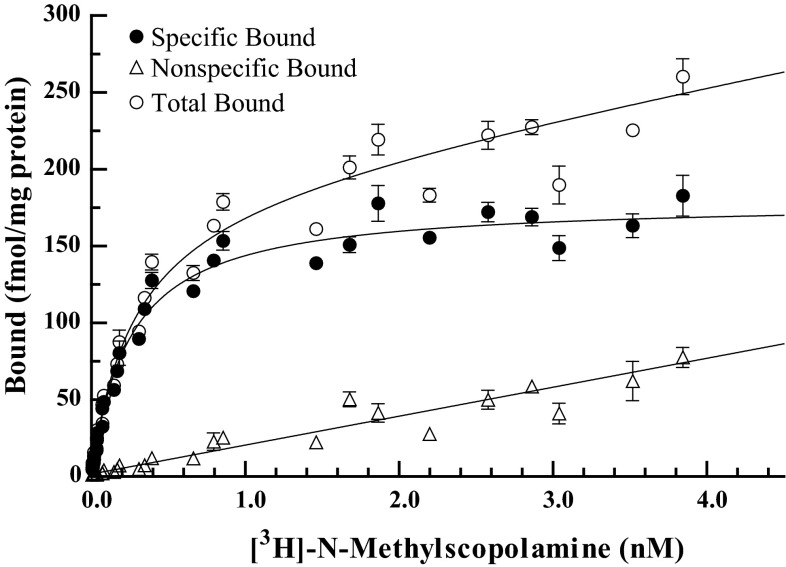
Receptor saturation binding studies with [^3^H]-NMS as the radioligand performed on African naked mole-rat spinal cord tissue. Non-specific binding (*unfilled up pointed triangle*), total binding (*unfilled circle*) and specific binding (*filled circle*) are shown. Data points are mean (±SEM) of three pooled saturation binding studies each conducted in triplicate and are corrected for protein content

With the amount of African naked mole-rat spinal cord tissue available, three receptor saturation binding assays using [^3^H]-NMS were performed. The pooled data are shown in Fig. 4. The mean (±SEM) *K*_d_, *B*_max_ and *n*_H_ values for [^3^H]-NMS binding to mAChRs in naked mole-rat spinal cord were: 309 ± 55 pM, 186 ± 7 fmol/mg protein and 1.08 ± 0.05, respectively.

## Discussion

Opioids and non-steroidal anti-inflammatory drugs (NSAIDs) are essential when it comes to treating moderate to severe pain (Angst and Clark 2006), but adverse effects are seen with both (Kaminaga et al. 1999; Pergolizzi et al. 2012). In addition, opioids are suggested to cause opioid-induced hyperalgesia (OIH), tolerance development to the drug, and potential opioid addiction (Angst and Clark 2006; Chu et al. 2006; Pergolizzi et al. 2012). Hence, there is a need for developing analgesic drugs that affect other receptor systems that the opioid.

Neurons with cholinergic receptors in the spinal cord terminate both at primary afferent fibers (PAF) and on intrinsic neurons like projection neurons, where they have the potential to modulate nociceptive information from both these types of neurons (George et al. 1962; Harris et al. 1969; Ireson 1970; Bartolini et al. 1987, 2011; Caulfield and Birdsall 1998). The suggested antinociception-mediating effects for the muscarinic receptor subtypes M_1_ and M_4_ makes them interesting potential drug targets for pain relief, but there is still a considerable lack of knowledge about the specific mechanism by which the muscarinic receptors exert their antinociceptive effect.

By studying different mammalian species with known differences in nociceptive behavior, further knowledge of the fundamental mechanisms involved in muscarinic regulation of antinociception can be obtained. In the present investigation, we have studied muscarinic receptors in the African naked mole-rat, with regards to the antinociceptive effects of muscarinic receptor ligands in vivo, as well as the pharmacological properties of their muscarinic receptors in vitro.

As mentioned, the naked mole-rat has some unusual properties regarding its pain physiology. It has been shown that naked mole-rats develop hyperalgesia when administered opioid agonists and then subjected to the hot-plate test (Towett et al. 2006). This response is similar to what is seen in chronic pain patients treated with opioids who develop opioid-induced hyperalgesia (Angst and Clark 2006; Chu et al. 2006). In addition, the animal has a complete lack of cutaneous C-fibers immunoreactive to substance P and calcitonin gene-related peptide (Park et al. 2003, 2008).

To the best of our knowledge, the mAChRs have not been investigated in the African naked mole-rat, except for one prior study (Dulu et al. 2014). Thus, the focus of this study was to further investigate the presence and function of mAChRs in the naked mole-rat, with the main focus on the mAChR subtypes M_1_ and M_4_.

Using BLAST it was found that the *m*_*1*_ and *m*_*4*_ receptors of the house mouse (*Mus musculus*), both were found to have a maximal identity of 95 % with *m*_*1*_ and *m*_*4*_ of the naked mole-rat. In both cases the total score was the same as the maximal score (652 for *m*_*1*_ and 903 for *m*_*4*_), and the query cover was found to be 81 and 100 % for the *m*_*1*_ and *m*_*4*_ sequences, respectively. This shows that the naked mole-rat has genes coding for proteins with high similarity to mAChRs in the house mouse.

As shown in earlier studies (Kanui et al. 1993; Park et al. 2008; Towett et al. 2009; Dulu et al. 2014), the formalin test is a reliable nociceptive test in the naked mole-rat, which was confirmed in this study. The administration of 8.4 mg/kg (s.c.) xanomeline resulted in a significant decrease of pain behavior during the late phase. In the experiment with 8.4 mg/kg xanomeline co-administered with 2.5 mg/kg atropine, a significant difference was found in the late phase when compared to the administration of 8.4 mg/kg xanomeline alone. The effects of xanomeline are suggested to be mediated through binding to the mAChRs M_1_ and/or M_4_ (Martino et al. 2011), which indicates that these subtypes should be of importance for the effects observed. However, since the actual concentration of xanomeline at the site of receptors in the present study is unknown, it cannot be ruled out that xanomeline exerts some of its actions through other mAChR subtypes than M_1_ and M_4_. For instance, xanomeline has also been described as an M5 receptor antagonist (Grant and El-Fakahany 2005), which could contribute to the observed effects.

When administering 28.1 mg/kg of xanomeline, it was found that the activity of the animals was markedly decreased about 20 min after administration and, as mentioned above, most of the animals given the 28.1 mg/kg dose had reduced proprioception and motor skills after the formalin test had been conducted. This could be related to several factors. However, the reduced proprioception and motor skills were reversed in animals treated with 28.1 mg/kg xanomeline in co-administration with 2.5 mg/kg atropine. This suggests that mAChRs, at least partly, mediate the cause of the reduced motor functions.

In this study, administration of 50 mg/kg of the M_4_ specific allosteric agonist VU0152100 did not produce a significant decrease in pain behavior, although a tendency was observed from 35 min and lasting throughout (Fig. 3). In addition, a significant difference was found between co-administration of VU0152100 with 2.5 mg/kg atropine and VU0152100 alone during the late phase. This hints towards a possible antinociceptive action of VU0152100 mediated through mAChRs, possibly M_4_.

In none of the experiments with xanomeline and VU0152100, a significant decrease in pain behavior during the early phase was observed, contrary to what was observed in a previous study using the nonspecific mAChR agonist oxotremorine in the formalin test (Dulu et al. 2014). In that study the drugs were administered i.p. 30 min prior to the formalin test, similar to this study with xanomeline and VU0152100. Therefore, neither animal species nor administration route and time of administration are likely the cause of the lack of early phase response the present study. A possible explanation for the differences observed in antinociception could be different pharmacokinetic profiles of these drugs in the naked mole-rat. Other possible explanations to the absent effect in the early phase could be different stress level in the animals, or variation between stocks of animals used in the two studies.

Since an antinociceptive effect of xanomeline similar to that of the naked mole-rat was also reported in mice and rats (Sheardon et al. 1997; Martino et al. 2011), and since the mole-rat has genetic and pharmacological similarities regarding mAChRs, it is assumable that the mechanisms of action for the antinociceptive effect of mAChRs are similar in the naked mole-rat and the laboratory mouse. Hence, it seems that the behavioral and physiological differences in the mole-rats pain system that have been previously observed are not related to the mAChR-system.

The saturation binding assay data demonstrates that mAChRs are present and have pharmacological function in the naked mole-rat. The precise location of the receptors in the spinal cord is, however, not known. This means that we cannot conclude if the effects of xanomeline and VU0152100 occur by action on excitatory or inhibitory interneurons in the spinal cord, on projection neurons, or on primary afferent neurons. This could be elucidated by studying immunohistochemistry of spinal cord tissue from the naked mole-rat. However, since we conclude that the muscarinic receptor system of the naked mole-rat des not account for the differences in pain behavior compared to other rodents, we choose not to proceed with this matter in the present study. For the same reason, we choose not to investigate the precise composition of mAChR subtypes by the use of specific receptor subtypes such as pirenzepine (M_1_ antagonist), AF-DX 116 (M_2_ receptor antagonist), 4-DAMP (M_3_ receptor antagonist) and tropicamide (M_4_ receptor antagonist). Hence, this was beyond the scope of the present study. However, it would certainly be of great interest to investigate this in future studies, to fully characterize the muscarinic receptor system in the naked mole-rat.

It must also be pointed out since xanomeline and VU0152100 were administered systemically, the antinociceptive actions of the drugs are necessarily not solely located solely in the spinal cord. Furthermore, it must also be pointed out that the number of publications where xanomeline or VU152100 have been studied are scarce, why the site of action of these substances should be considered unclear for any species at this stage.

In conclusion, BLAST analysis showed that the African naked mole-rat has genes coding for proteins similar to all five mAChR subtypes. The results from the formalin test suggest the antinociceptive effects of xanomeline and VU0152100 to be mediated through mAChRs. A significant reduction in pain behavior was seen after administration of 8.4 and 28.1 mg/kg xanomeline and the effect was reversed by atropine, an mAChR antagonist. The reduction in pain behavior after VU0152100 administration was not significant, but a trend towards antinociception was seen, which was reversed by atropine. The receptor saturation binding study performed with [^3^H] N-methylscopolamine on spinal cord tissue from the African naked mole-rat resulted in saturable specific binding, but it was not possible to determine to which mAchR subtype(s). These data therefore suggest that mAChRs are present in the African naked mole-rat and that they contribute to the regulation of pain transmission, but that further investigations are needed to give more definitive answers regarding the distribution and function of mAChR subtypes M_1_ and M_4_ in the naked mole-rat.
